# Supplementary material for: Personal transcriptome variation is poorly explained by current genomic deep learning models
Source: Nat Genet. 2023 Nov 30;55(12):2056–9. doi: 10.1038/s41588-023-01574-w (PMC10703684; doi:10.1038/s41588-023-01574-w)
Supplement: Supplementary file 1 — Supplementary Figs. 1–5. [file 41588_2023_1574_MOESM1_ESM.pdf]

nature genetics

Brief Communication

https://doi.org/10.1038/s41588-023-01574-w

Personal transcriptome variation is poorly explained by current genomic deep learning models

In the format provided by the
authors and unedited

Supplementary Figures

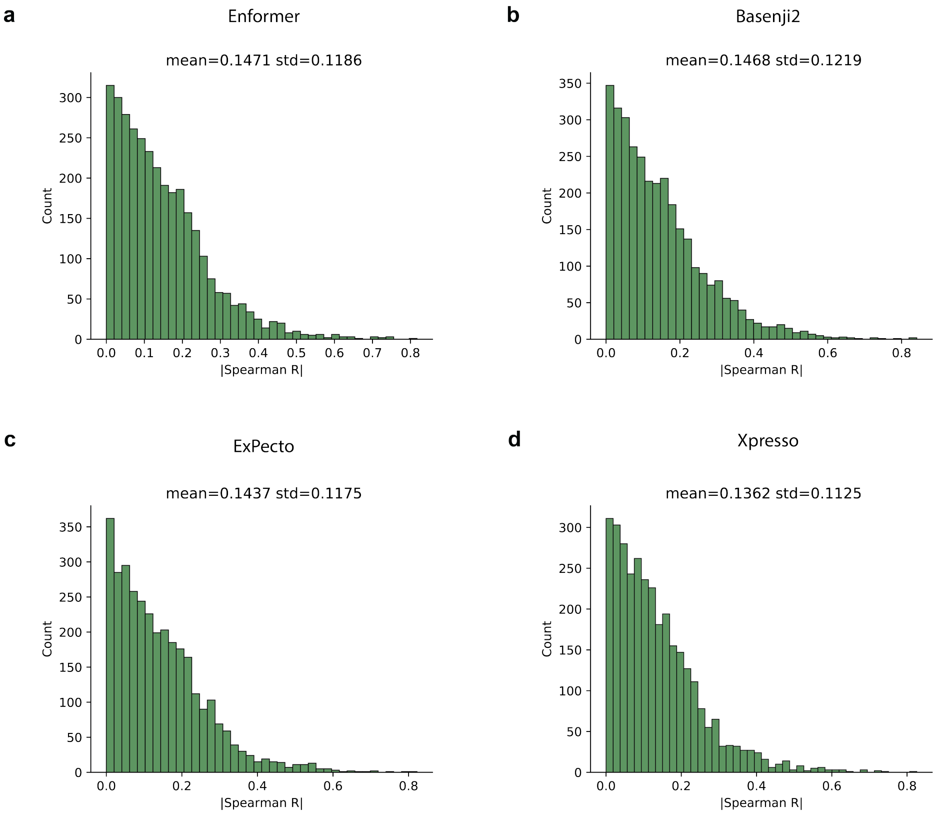

Supplementary Fig. 1: **Absolute values of cross-individual correlations.** Absolute value distributions of cross-individual performance for (a) Enformer, (b) Basenji2, (c) ExPecto, and (d) Xpresso. Cross-individual performance is computed as in Extended Data Fig. 3. Each histogram displays the distribution of the absolute value of the cross-individual performance for the 3,259 genes with at least one statistically significant (FDR $< 5\%$) eQTL in the Geuvadis analysis.

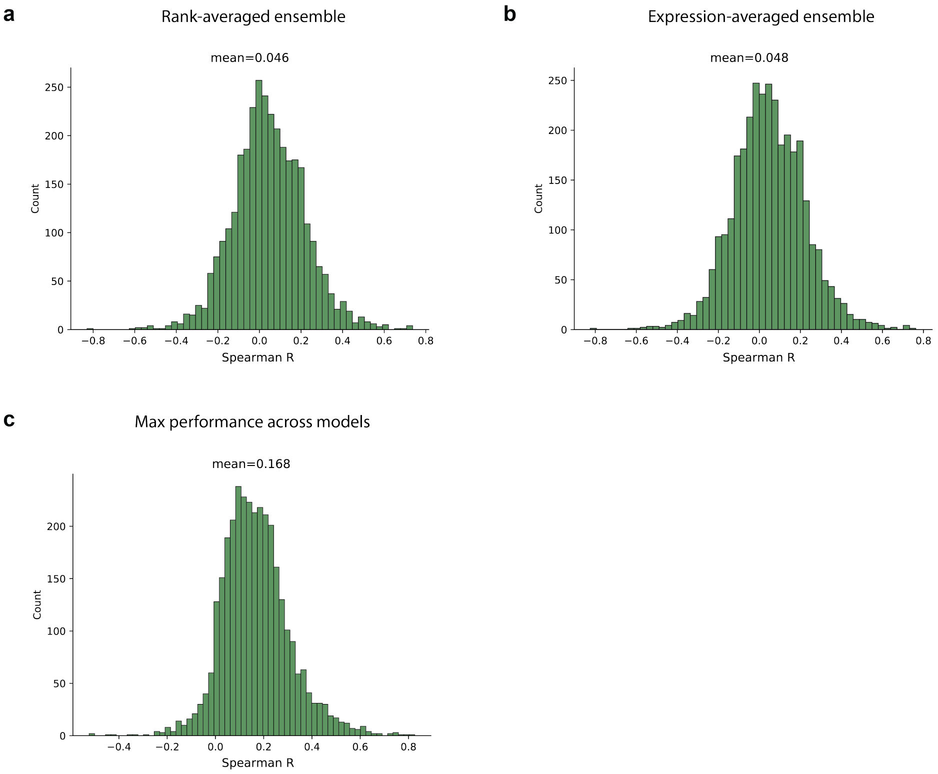

Supplementary Fig. 2: **Cross-individual performance of ensemble models.** Predictions from the four deep learning models (Enformer, Basenji2, ExPecto, Xpresso) were ensembled per gene per individual by averaging (a) the z-scored expression predictions or (b) the cross-individual expression ranks from each model. For a given gene, cross-individual performance is defined as the correlation between measured gene expression levels in all 421 individuals and corresponding gene expression predictions from the ensemble models. Each histogram displays the distribution of cross-individual performance for the 3,259 genes with at least one statistically significant (FDR < 5%) eQTL in the Geuvadis analysis. In (c), we plot the distribution of the maximum cross-individual performance (Spearman R) per gene across the four deep learning models (from Extended Data Fig. 3).

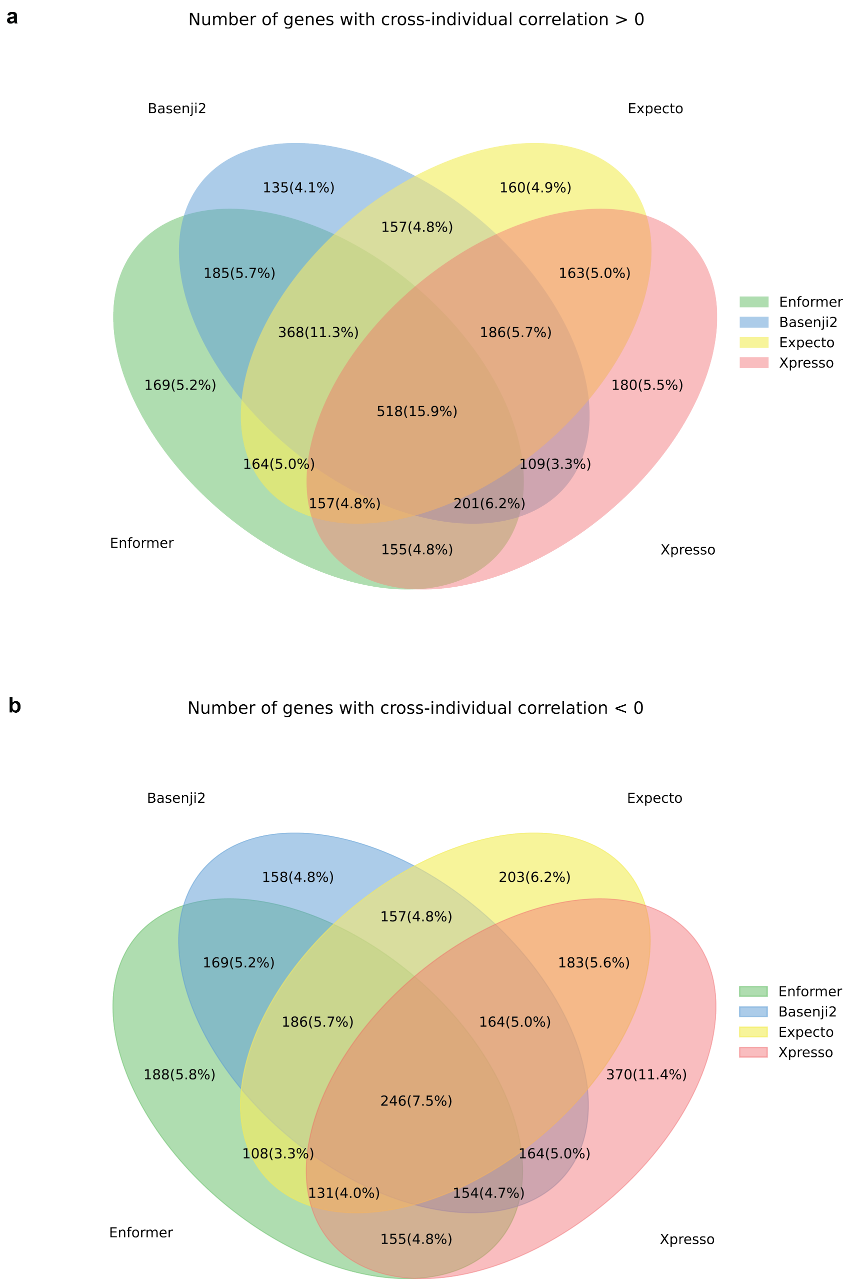

Supplementary Fig. 3: **Consistency among methods.** Overlap between the four tested deep learning models in (a) genes with a positive cross-individual correlation and (b) genes with a negative cross-individual correlation. Percentages are out of the 3,259 genes with at least one statistically significant (FDR < 5%) eQTL in the Geuvadis analysis.

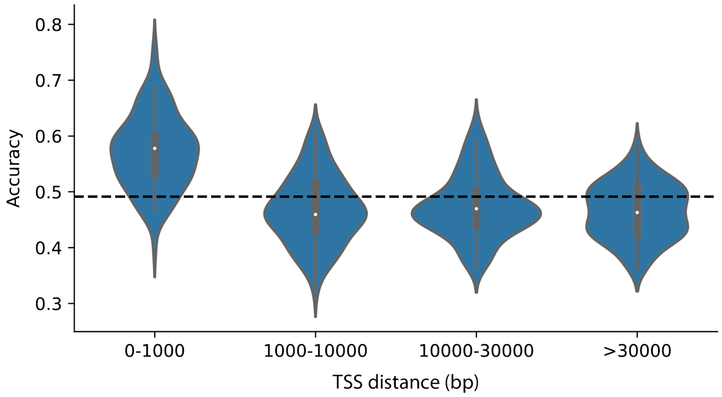

Supplementary Fig. 4: **Enformer performance on predicting direction of effect for fine-mapped eQTLs.** Enformer expression direction prediction accuracy for GTEx EBV-transformed lymphocyte cell eQTLs fine-mapped using SuSiE. Enformer predictions from the GM12878 CAGE track were used to predict whether the minor allele of each fine-mapped eQTL increases or decreases gene expression. Accuracy on this task is stratified across four bins of variant distance to TSS. Violin plots show the accuracy distribution over 100 bootstrap samples from the full set of variants. The mean accuracy over all distances is also shown (dashed line).

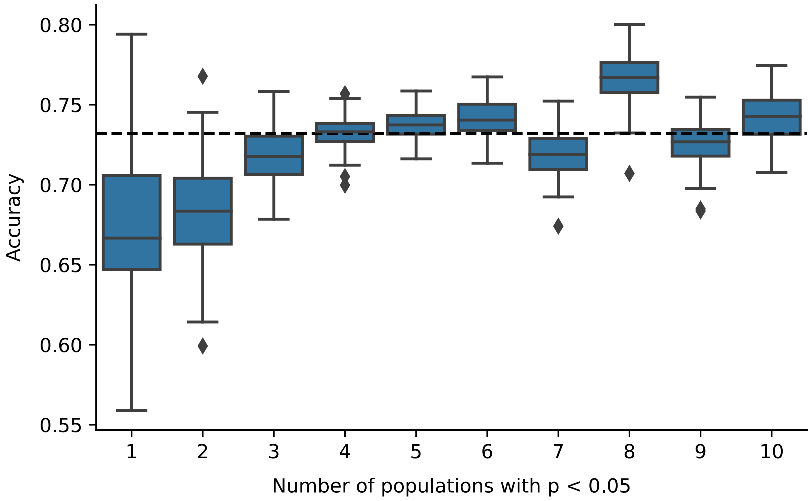

Supplementary Fig. 5: **Enformer performance on predicting direction of effect for caQTLs.** Enformer's accuracy at classifying alternate alleles as more or less accessible than reference, using variants from the Tehranchi *et al.* dataset in which chromatin accessibility quantitative trait loci (caQTLs) in LCLs were called from ATAC-seq data. Accuracy on this task is stratified by the number of populations (out of ten: four African, four European, one African-American, and one Han Chinese) in which the caQTL was identified as statistically significant with $p < 0.05$. Boxplots show the accuracy distribution over 100 bootstrap samples of the data. The center line indicates median, boxes extend from first to third quartiles, whiskers show the data range (up to 1.5 times the interquartile range from the box edges), and diamonds indicate outliers. The mean accuracy over all bins is also shown (dashed line). Note that these accuracies should not be compared directly with those for finemapped eQTLs reported in Supplementary Fig. 4 because of substantial differences in the two methods for calling QTLs.
